# Supplementary material for: Robust High-Throughput Phenotyping with Deep Segmentation Enabled by a Web-Based Annotator
Source: Plant Phenomics. 2022 May 18;2022:9893639. doi: 10.34133/2022/9893639 (PMC9394117; doi:10.34133/2022/9893639)
Supplement: Supplementary Materials — Figure S1. Example predictions on Pascal VOC dataset on a single click. Figure S2. Example predictions on Pascal VOC dataset on two clicks. Figure S3. Example predictions on Pascal VOC dataset on three clicks. Figure S4. Example predictions on Leaf Segmentation Challenge (LSC) dataset on two clicks. Figure S5. Example predictions on Leaf Segmentation Challenge (LSC) dataset on three clicks [file 9893639.f1.pdf]

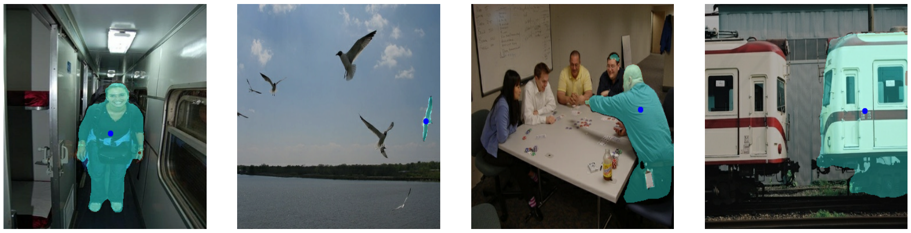

Click 1

Figure S1. Example predictions on Pascal VOC dataset on a single click.

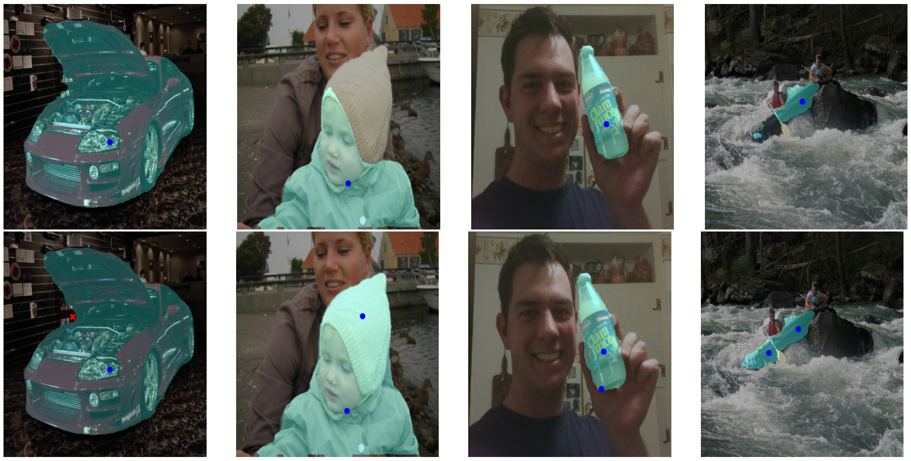

Click 1

Click 2

Figure S2. Example predictions on Pascal VOC dataset on two clicks.

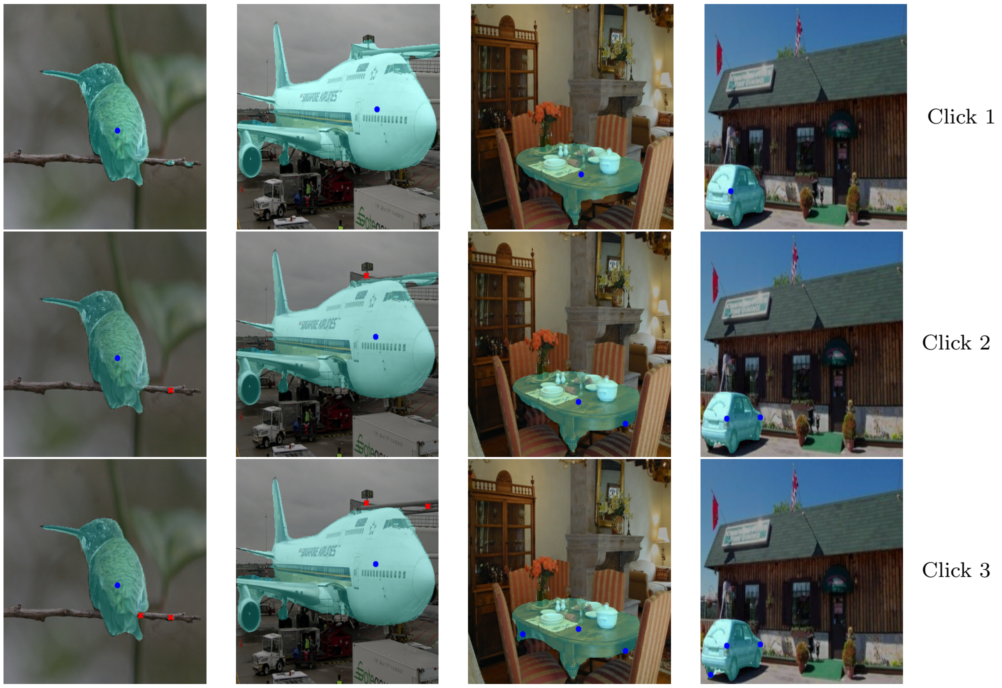

Figure S3. Example predictions on Pascal VOC dataset on three clicks.

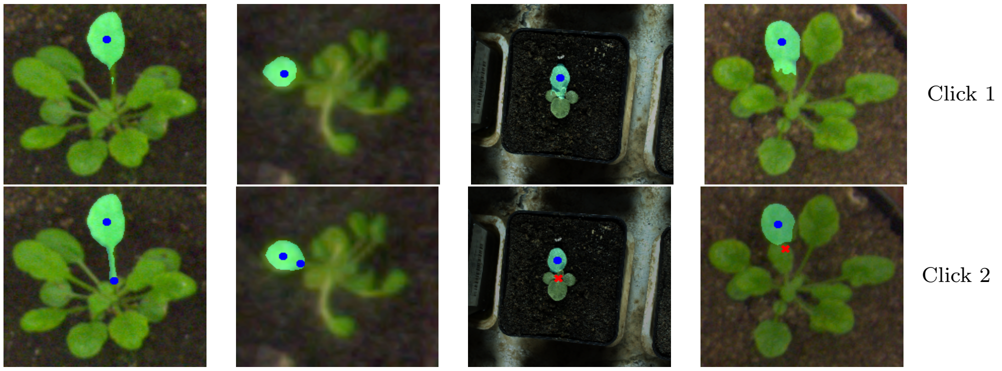

Figure S4. Example predictions on Leaf Segmentation Challenge (LSC) dataset on two clicks.

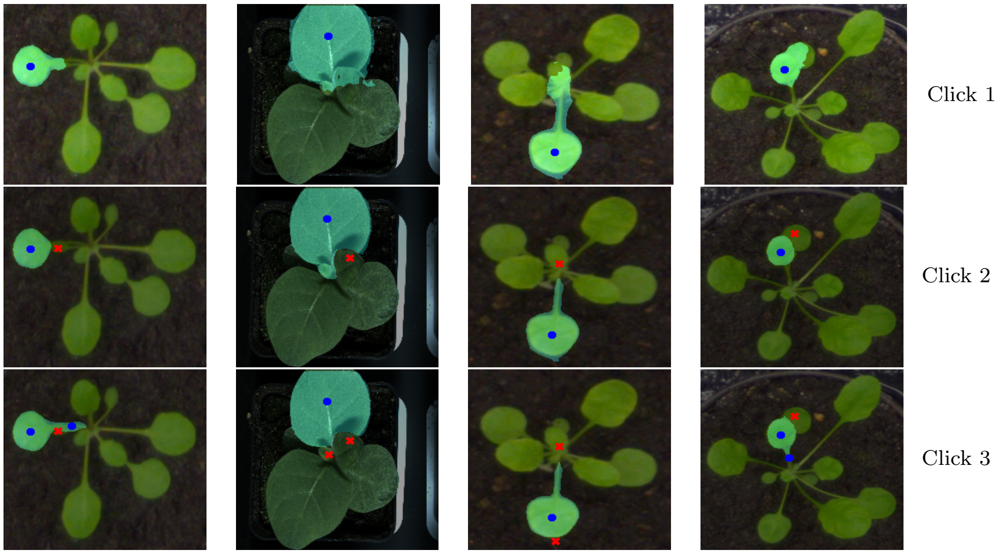

Figure S5. Example predictions on Leaf Segmentation Challenge (LSC) dataset on three clicks.
